# Clinical Neuropathology practice guide 4-2013: post-herpes simplex encephalitis: N-methyl-D-aspartate receptor antibodies are part of the problem 

**DOI:** 10.5414/NP300666

**Published:** 2013-07-04

**Authors:** Romana Höftberger, Thaís Armangue, Frank Leypoldt, Francesc Graus, Josep Dalmau

**Affiliations:** 1 Institute of Neurology, Medical University of Vienna, Austria,; 2 Service of Neurology, Hospital Clínic, Universitat de Barcelona and Institut d´Investigació Biomèdica August Pi i Sunyer (IDIBAPS), Barcelona, Spain,; 3 Department of Neurology, University Medical Center Hamburg-Eppendorf, Hamburg, Germany,; 4 Institució Catalana de Recerca i Estudis Avançats (ICREA), IDIBAPS, Hospital Clínic, Barcelona, Spain, and; 5 Department of Neurology, University of Pennsylvania, Philadelphia, PA, USA

**Keywords:** NMDAR antibodies, herpes simplex encephalitis, choreoathethosis, post-herpes simplex encephalitis

## Abstract

Classic herpes simplex virus encephalitis (HSVE) is an acute viral infection that usually follows a monophasic disease course; however some patients, mainly children, experience a relapse within weeks or months after the initial event. In a subset of these patients a viral reactivation is unlikely because the CSF PCR for HSV is negative, repeated MRI does not show new necrotic lesions, and the symptoms are refractory to antiviral therapy. These patients often develop choreoathetosis variably accompanied by behavioral changes and seizures, and a postinfectious immune-mechanism has been postulated. Recent studies demonstrated that 7% of patients with HSVE harbor NR1 N-methyl-D-aspartate receptor (NMDAR) IgG antibodies. Moreover, a child with post-HSVE choreoathetosis was found to have NMDAR antibodies; the patient did not improve with antiviral therapy but recovered after aggressive immunotherapy. Based on these findings, evidence is increasing that a subgroup of post-HSVE represents a separate disease entity, which in fact is anti-NMDAR encephalitis. Patients with relapsing HSVE or prolonged atypical symptoms, who have negative CSF PCR for HSV should routinely be tested for NMDAR IgG antibodies in CSF and serum. It is important to be aware of this differential diagnosis because patients respond to immunotherapy.

## Background 

Herpes simplex virus encephalitis (HSVE) is the most common non-epidemic form of viral encephalitis in Western countries [1]. The infection usually affects the limbic structures resulting in seizures, personality change, memory dysfunction and focal neurological deficits. The diagnosis is made by positive HSV polymerase chain reaction (PCR) in the cerebrospinal fluid (CSF) and patients often respond to anti-viral treatment. The disease usually follows a monophasic course, but 14 – 27% of the patients, often children, develop a recurrent encephalitic episode after successful treatment of the initial infection [2, 3, 4]. The pathogenesis of these relapses is heterogeneous (Table 1): some cases represent true relapses of viral encephalitis, with positive HSV PCR in the CSF, new necrotic lesions in the MRI, and response to antiviral treatment. In these patients the relapsing symptoms represent a reactivation of the viral replication, or delayed symptoms of a persistent infection [2, 3, 4, 5, 6, 7, 8, 9, 10, 11, 12, 13, 14, 15]. In contrast, in a subset of relapsing patients the mechanisms that initiate the disorder are less clear. Children frequently have dyskinesia and choreoathetosis that typically develop 4 – 6 weeks after the initial HSVE episode. In adult relapse cases, cognitive and psychiatric symptoms are more prominent and movement disorders have not been described [13, 16]. The CSF PCR for HSV is no longer positive, the MRI does not show new necrotic lesions, and symptoms do not respond to antiviral therapy. The exact etiology of this disorder has been unknown, but reports of patients who responded to immunotherapy suggested an immune-mediated pathogenic mechanism [2, 5, 13, 16, 17, 18, 19, 20, 21, 22, 23, 24, 25, 26, 27, 28, 29]. 

## New evidence for NMDAR antibodies in post-HSVE 

The hypothesis that a subgroup of non-infectious post-HSVE could have an immune-mediated pathogenesis has been recently supported by two studies discussed below, which indicate a link with anti-NMDAR encephalitis. 

Anti-NMDAR encephalitis is a subacute, severe, but potentially treatable autoimmune encephalitis defined by the presence of IgG antibodies against cell surface epitopes of the NR1 subunit of the NMDAR. The resulting syndrome is characterized by prominent change of behavior, psychosis, memory deficits, seizures, abnormal movements, coma and autonomic dysfunction [30, 31, 32]. Some patients, mainly young women, harbor an underlying teratoma (usually in the ovary), in others the triggering factor for the NMDAR antibody production is unknown. Prodromal symptoms such as headache, fever, diarrhea or upper respiratory symptoms are frequently reported, leading to the hypothesis that an infectious disease could trigger the immunological disorder. However, routine serological and CSF studies in many patients, and several studies examining possible viral triggers did not identify a specific infectious agent [33, 34]. Recently, IgG NMDAR antibodies, identical to those associated with anti-NMDAR encephalitis (targeting the NR1 subunit of the NMDAR), were detected in 7% of patients with HSVE [35]. This study suggested that some atypical symptoms following HSVE, including prolonged abnormal movements (not responsive to viral therapies) or even episodes of post-HSVE (e.g., choreoathetosis post-HSVE) could be related to anti-NMDAR antibodies, representing in fact, anti-NMDAR encephalitis. Indeed, a recent pediatric series on anti-NMDAR encephalitis included a patient with post-HSVE choreoathetosis who had serum and CSF IgG antibodies against the NMDAR and responded to intensive immunotherapy [17]. Due to the retrospective nature of the study, serum and CSF from the time of the viral infection were not available and therefore the time course of antibody synthesis was unclear. However, in a more recent observation of post-HSVE in an adult, NMDAR antibodies could not be detected in serum or CSF at presentation of viral encephalitis, but were detected several weeks later when the patient developed relapsing neurological symptoms, including change of behavior, psychosis and memory deficits. Analysis of CSF for HSV was no longer positive, and the patient responded well to immunotherapy, along with a decrease of NMDAR antibody titers (Leypoldt et al., personal observation). 

## Possible pathogenetic mechanisms 

These studies and observations offer new evidence of the occurrence of postviral autoimmunity against a known synaptic receptor. However, the question remains, which mechanisms specifically cause the breach of tolerance following HSVE. One possibility is molecular mimicry, whereby the viral protein sequence triggers an immune response that is misdirected against a structurally similar epitope present in the NMDAR. To date, there are no reports of a shared epitope sequence between HSV and NMDAR; future studies should address this possibility. Alternatively, the HSV-induced intense inflammatory response in limbic structures, usually accompanied by necrosis, could release and appropriately present abundantly expressed local NMDAR epitopes to the immunological system, breaking tolerance and initiating an autoimmune response. In this case, it would not be surprising that antibodies against other synaptic or neuronal cell surface antibodies might be identified in future studies. These could account for a wider spectrum of symptoms beyond the syndrome that frequently characterizes anti-NMDAR encephalitis [19]. 

## Conclusion 

In patients with relapsing symptoms of HSVE whose CSF PCR studies are negative for HSV routine diagnostic work-up should include screening for NMDAR antibodies in CSF and serum. Detection of these antibodies indicates that the disorder is anti-NMDAR encephalitis and should be treated with immunotherapy. 

## Acknowledgments 

The work was supported in part by the National Institutes of Health R01NS077851, R01MH094741, the National Cancer Institute R01CA089054, Fondo de Investigaciones Sanitarias (FIS, 11/01780), and Fundació la Marató de TV3 (Josep Dalmau), grant FIS PS09/0193, Madrid, Spain (Francesc Graus). RH was funded by the Fonds zur Förderung der wissenschaftlichen Forschung, Austria, Project J3230. FL was funded by the Forschungsförderungsfonds University Hospital Hamburg Eppendorf. 

Dr. Dalmau has a research grant from Euroimmun, and receives royalties from patents for the use of Ma2 and NMDAR as autoantibody tests. Dr. Leypoldt has received speakers honoraria from Grifols and scientific funding from Euroimmun. Drs. Höftberger, Armangue and Graus declare no conflict of interest. 

**Table 1.** Post-HSVE: clinical features related to two pathogenic mechanisms.

	Infectious post-HSVE	Autoimmune post-HSVE
Median age in years; (range)^a^	5.25 (0.3 – 71)	3 (0.3 – 67)
Male : female^a^	15 : 8	12 : 7
Neurological symptoms^a^	Focal neurological signs, seizures, behavioral abnormalities, disorientation; three cases with choreoathetosis [5, 6, 8]	Choreoathetosis, ballism; one case with personality change, sleep disorder and bulimia [19];
Time from initial HSV infection to relapsing symptoms	Variable	4 – 6 weeks
HSV PCR in CSF	Positive	Negative
New necrotic lesions on MRI	Yes	No
Response to anti-viral therapy	Yes	No
Etiology	Infectious	Autoimmune

^a^Based on review of the literature; cases considered by the authors as infectious HSVE relapses (n = 28; age available in n = 26; gender available in n = 23) [2, 3, 4, 5, 6, 7, 8, 9, 10, 11, 12, 13, 14, 15] and autoimmune mediated HSVE relapses (n = 33; age available in n = 23; gender available in n = 19) [2, 5, 13, 16, 17, 18, 19, 20, 21, 22, 23, 24, 25, 26, 27, 28, 29].
